# Olfaction in alcohol-dependence: a neglected yet promising research field

**DOI:** 10.3389/fpsyg.2013.01007

**Published:** 2014-01-03

**Authors:** Pierre Maurage, Philippe Rombaux, Philippe de Timary

**Affiliations:** ^1^Laboratory for Experimental Psychopathology, Psychological Sciences Research Institute, Université catholique de LouvainLouvain-la-Neuve, Belgium; ^2^Department of Otorhinolaryngology, St Luc Hospital and Institute of Neuroscience, Université catholique de LouvainBrussels, Belgium; ^3^Department of Adult Psychiatry, St Luc Hospital and Institute of Neuroscience, Université catholique de LouvainBrussels, Belgium

**Keywords:** olfaction, cognition, emotion, alcohol-dependence, orbitofrontal cortex, executive functions

## Abstract

Olfaction research deeply renewed the knowledge of the pathophysiological mechanisms involved in various psychopathological states and showed that olfactory deficits might constitute an onset or trait marker in psychiatry. However, while alcohol-dependence is the most wide spread psychiatric disorder and while olfaction might be involved in its development and maintenance, olfactory abilities have been little explored in this population. The central aim of this paper is thus to underline the usefulness of olfaction research in alcohol-dependence. After reviewing the few olfaction studies available, a research agenda will be proposed, identifying the major challenges for future research, and particularly: (1) the identification of the origin, extent and cerebral correlates of olfaction deficits; (2) the links between olfaction and emotional-cognitive deficits, and the use of olfaction to understand the pathomechanisms of alcohol-dependence; (3) the interactions between olfaction and other sensory modalities; (4) the use of olfaction to predict the appearance and intensity of cognitive impairments; (5) the impact of olfaction deficits on everyday life in alcohol-dependence.

## INTRODUCTION

Alcohol-dependence represents a major concern for public health, annually leading to more than two million deaths worldwide (World Health Organization [WHO], 2011). The excessive consumption of alcohol has deleterious physiological effects, notably on the central nervous system as alcohol-dependence is associated with cerebral impairments (see Bühler and Mann, 2011 for a review of more than 190 papers on brain correlates of alcohol-dependence). The consequences of these brain deficits on cognitive and emotional abilities have also been widely described: alcohol-dependent individuals present impaired performance in attentional, executive, or memory abilities (Stavro et al., 2013), but also in affective and interpersonal processing (Philippot et al., 1999; Maurage et al., 2012). This large amount of data now offers a nearly exhaustive view of the deleterious consequences of excessive alcohol consumption.

However, these numerous studies have nearly all be based on visual or auditory stimulations. This can be explained by the fact that vision and audition are the most frequently used sensorial modalities among humans and are also the easiest to implement in experimental settings. Nevertheless, the exclusive focus on two modalities led to the nearly total neglect of other senses, and particularly olfaction. This lack of data on olfactory abilities constitutes a major shortcoming for the understanding of alcohol-dependence, as odors might play a crucial role in the development and persistence of this addictive state. The main aim of the present paper will thus first be to underline this importance of olfactory processes in alcohol-related disorders. After reviewing the results of the few studies which explored olfaction in alcohol-dependent patients, we will then underline the usefulness of olfactory studies to offer a better understanding of the impairments presented in alcohol-dependence. Particularly, we will show how olfaction might deeply renew and improve the current knowledge about this pathology, at fundamental (e.g., by renewing the knowledge on the pathophysiological mechanisms involved) and clinical (e.g., by developing olfaction rehabilitation programs to improve quality of life) levels.

## WHY SHOULD OLFACTION BE EXPLORED IN ALCOHOL-DEPENDENCE?

There are at least four main arguments promoting further exploration of olfaction in alcohol-dependence:

### THE IMPACT OF OLFACTORY LOSS ON EVERYDAY LIFE

Olfactory impairments could influence the decreased quality of life observed in alcohol-dependence. Indeed, olfactory loss is deleterious for everyday activities (Shu et al., 2011) as it increases the risk of injury by hampering the identification of environmental hazards (Stevenson, 2010). It also lowers the richness of social life (Schiffman, 1997), as smells are involved in social choices (Li et al., 2007; Prehn-Kristensen et al., 2009). Olfaction impairments thus have major consequences on personal and social life. As odors are crucial for food enjoyment (Smeets et al., 2009) and regulation (Stevenson, 2010), altered olfaction might participate in abnormal feeding behaviors (Santolaria et al., 2000) and nutrition deficiencies reported in alcohol-dependence (Carey, 1989). Olfaction impairments might largely alter food choices or eating motivation in this population, which underlines the need to better understand these impairments.

### THE INTERACTIONS BETWEEN OLFACTION AND EMOTIONAL-COGNITIVE ABILITIES

The olfactory system is connected with cognitive and emotional brain regions, and exploring olfaction might improve the understanding of emotional-cognitive deficits in alcohol-dependence (up to now explored with visuo–auditory stimulations). Olfaction is indeed directly connected with limbic (Soudry et al., 2011) and fronto-temporal regions (Rolls, 2004). The orbitofrontal cortex is a crucial area in this perspective, being simultaneously involved in emotional, executive, and olfactory processing (Rolls, 2008). Strong correlations between olfactory and cognitive abilities have been shown (Purdon, 1998; Schubert et al., 2008; Sohrabi et al., 2012), underlining their common cerebral basis. Olfaction testing is also used to explore cognitive impairments in neurodegenerative disorders (Velayudhan et al., 2013). Olfaction thus constitutes an interesting way to renew the exploration of emotional-executive deficits in alcohol-dependence.

### THE PROMISING RESULTS OF OLFACTION STUDIES IN PSYCHIATRY

Odor-processing impairments have been described in schizophrenia (Strauss et al., 2010), autism (Wiggins et al., 2009), anorexia nervosa (Roessner et al., 2005), and depression (Clepce et al., 2010). Beyond the mere description of impaired olfactory abilities, olfaction research offered new fundamental insights on psychiatric states, notably on three aspects. First, it renewed the knowledge on the psychophysiological mechanisms involved [e.g., dopamine regulation in schizophrenia (Moberg et al., 2013; Schecklmann et al., 2013)]. Second, it proposed early diagnostic tool for neurodegenerative diseases [e.g., Alzheimer (Luzzi et al., 2007) or Parkinson disease (Kranick and Duda, 2008)] and schizophrenia (Brewer et al., 2003; Turetsky et al., 2008). Third, as the level of olfactory deficit varies across pathologies, olfaction might constitute a cognitive marker in psychiatry (Atanasova et al., 2008) and a reliable evaluator of disease severity (Rupp, 2010; Segalàs et al., 2011). However, while constituting a topic of rising importance in psychiatry, odor processing remains little investigated in alcohol-dependence.

### THE POSSIBLE ROLE OF OLFACTION IN THE DEVELOPMENT AND MAINTENANCE OF ALCOHOL-DEPENDENCE

Alcohol beverages provoke massive orthonasal and retronasal stimulations (Bragulat et al., 2008) which constitute strong appetitive cues (Bienkowski et al., 2004) and might be involved in the arisen of alcohol-dependence as they rapidly lead to conditioned alcohol-seeking behaviors (Pautassi et al., 2009). Olfactory stimulations elicit strong drinking desires (Schneider et al., 2001), this olfactory craving being even stronger than those provoked by visual–auditory cues, particularly during withdrawal (Kareken et al., 2004; Little et al., 2005) and thus being potentially involved in relapse. These preliminary results suggest that olfactory cues might play a role in the appearance of alcohol-dependence, but further studies are needed to explore their role at different stages of dependence.

## WHAT IS CURRENTLY KNOWN ABOUT OLFACTION DEFICITS IN ALCOHOL-DEPENDENCE?

Earlier studies used olfactory cues in alcohol-dependence to elicit a consumption urge, and showed that alcohol-related odors can provoke strong subjective and physiological craving responses (Stormark et al., 1995; Bordnick et al., 2008). Neuroimaging studies showed that odor-induced craving mostly relies on a limbic and reward network including nucleus accumbens (Kareken et al., 2004), amygdala–hippocampal (Schneider et al., 2001), and orbitofrontal (Bragulat et al., 2008) regions. Nevertheless, as they were designed to test whether odors elicit craving, these studies did not explore odor processing and brought no insight on olfaction impairments.

Few studies directly explored olfaction in alcohol-dependence. The initial explorations led to contradictory results, some showing impaired odor discrimination, identification, or recall (Potter and Butters, 1979; DiTraglia et al., 1991) while others described preserved olfaction (Jones et al., 1975, 1978; Mair et al., 1986; Kesslak et al., 1991). These discrepancies might be explained by large methodology and population variations, and by the lack of control for medication and comorbidities. More recently, the use of a validated battery separately exploring three olfaction abilities [odor detection threshold, discrimination, identification (Kobal et al., 2000)] allowed to show a generalized olfactory deficit in alcohol-dependence, independent of medication and smoking habits (Rupp et al., 2003), as well as impaired familiarity and edibility odor judgments (Rupp et al., 2004). As olfactory judgments largely rely on orbitofrontal cortex (Royet et al., 2001), these results support the involvement of this region in olfaction deficits. However, this hypothesis had to be confirmed by studies including neuroimaging exploration or other cognitive tasks testing orbitofrontal cortex.

To explore this orbitofrontal implication, a simultaneous exploration of odor processing and executive functions was subsequently proposed (Rupp et al., 2006). Results confirmed olfaction impairments and showed a strong correlation between odor discrimination and executive performance, suggesting that orbitofrontal cortex is involved in olfactory impairments. Nevertheless, these executive tasks did not specifically rely on orbitofrontal functioning but rather on a large frontal network. In line with these results, we recently explored these olfaction-executive links by simultaneously administrating a complete odor processing test, the confabulation task [a source memory test specifically involving the orbitofrontal cortex (Schnider et al., 1996)] and another executive task unrelated to orbitofrontal cortex. Alcohol-dependence was associated with impaired odor identification and source memory, but preserved non-orbitofrontal performance. Centrally, a strong correlation was found between olfaction and source memory performances, suggesting that both abilities rely on orbitofrontal cortex (Maurage et al., 2011a).

Finally, only two studies directly explored the cerebral correlates of olfaction in alcohol-dependence. A MRI study showed a correlation between olfaction deficits and decreased cerebral volumes in a large cortico-subcortical network (Shear et al., 1992). More recently, we explored the electrophysiological consequences of alcohol-dependence on olfaction (Maurage et al., 2011b) using chemosensory event-related potentials. Results showed altered olfactory event-related potentials (related to smell and indexing olfactory nerve activation) in alcohol-dependence, with preserved trigeminal activity (related to nasal somatosensory feelings and indexing trigeminal nerve activation). This shows that the olfactory deficit is specific and not due to a general impairment also affecting trigeminal functioning. Moreover, the electrophysiological deficit was mostly present for the P2 wave (a high-level cognitive component related to endogenous cortical olfactory processing) with partial preservation of the N1 wave (indexing low-level sensory processing linked with the exogenous activity provoked by the odor). This suggests that olfaction deficits rely on impaired high-level cognition based on fronto-limbic network (including orbitofrontal cortex and amygdala) and not on low-level olfactory processing in primary olfactory cortex.

## SEVEN CRUCIAL QUESTIONS FOR FUTURE RESEARCH

Earlier studies presented above suggest that odor processing modifications might influence the everyday life of alcohol-dependent patients and claim for further exploration of olfaction deficits. Seven of the central questions that should be addressed will now be described, drawing a potential research agenda:

### WHAT ARE THE ORIGIN AND EXTENT OF OLFACTION DEFICITS?

The presence of olfaction impairments is established, but controversial results were obtained across earlier studies concerning the respective impairment of different olfactory abilities. Variations in alcohol-dependence’s characteristics (e.g., duration/severity of alcohol-dependence, duration of abstinence) might explain this controversy, and future studies should evaluate olfactory sub-components on larger populations to determine which factors modulate the impairment. A second step is to further explore other olfactory functions and particularly high-level odor judgments, which appear impaired (Rupp et al., 2004). Future studies should determine the preserved/impaired abilities, notably by using more subtle tests (Delplanque et al., 2008). As earlier studies focused on recently detoxified patients, the evolution of olfactory impairments across the successive stages of alcohol-dependence should also be explored. Cognitive functions recover with abstinence (Pitel et al., 2009), and testing olfaction at several stages of abstinence would determine how this deficit evolves when alcohol consumption stops. Finally, olfactory impairments are supposed to be provoked by alcohol-dependence, but this causal link should be tested. Studies in populations at-risk for schizophrenia showed that olfactory deficit precedes the pathology (Turetsky et al., 2008). Accordingly, exploring olfaction in populations at-risk for alcohol-dependence would determine if olfaction impairments mostly precede or follow alcohol-dependence.

### WHAT ARE THE CEREBRAL BASES OF THE DEFICIT?

While the brain correlates of olfactory impairments have been explored in clinical populations (Turetsky et al., 2003; Welge-Lüssen et al., 2009), underlining the key role of orbitofrontal cortex (Frasnelli et al., 2010; Li et al., 2010), only two studies investigated this question in alcohol-dependence (Shear et al., 1992; Maurage et al., 2011b). Behavioral explorations suggested an implication of the orbitofrontal cortex in olfactory dysfunctions but no neuroimaging data confirmed it. A combined neuroscience approach could identify these cerebral impairments and particularly the processing level at which olfactory deficits begins. Indeed, if the deficit is, as suggested (Maurage et al., 2011b), focused on high-level cognitive stages (limbic areas, orbitofrontal cortex) with preserved low-level sensory ones (olfactory bulb, primary olfactory cortex), therapeutic programs should focus on these high-level functions. More globally, neuroimaging studies of olfaction would complement the knowledge on the cerebral consequences of alcohol-dependence, currently based on visuo–auditory modalities. Another important issue is the contradiction between impaired processing (and reduced cerebral activations) for non-alcohol-related odors and increased cerebral activations for alcohol-related odors (Bragulat et al., 2008). Alcohol-dependence might lead to a double olfactory system modification: over-activation for alcohol-related cues, reduced activation for other stimuli. A better understanding of how motivational factors may persistently activate olfactive responses to some but not all olfactory stimuli would also give new insights on olfaction-motivation interactions.

### WHAT ARE THE LINKS BETWEEN OLFACTION AND EMOTIONAL-COGNITIVE FUNCTIONS?

Large-scale cognitive and emotional impairments have been described in alcohol-dependence using visual and auditory stimuli. As olfaction is the only modality possessing straightforward connections (Price, 1987) with emotional (amygdala) and cognitive (orbitofrontal cortex) areas, olfaction studies could renew the exploration of these emotion–cognition deficits. At the cognitive level, results suggesting links between olfaction and executive dysfunctions (Rupp et al., 2006; Maurage et al., 2011a) and showing that olfaction impairments might predict cognitive decline (Killgore et al., 2010) should lead to further explore these mutual influences. This is reinforced by the strong connections observed between olfaction, executive functions and memory in psychiatry (Murphy et al., 2001; Good et al., 2002). At the emotional level, the simultaneous implication of limbic structures in odor and affective processing (Soudry et al., 2011) and the olfaction–emotion interactions in healthy populations (Retiveau et al., 2004; Chrea et al., 2009) encourage the use of olfaction to explore emotional alterations. Moreover, odors strongly influence affective states (Lehrner et al., 2005; Moss et al., 2008, 2010), and applying these paradigms in alcohol-dependence might open new perspectives. Finally, as connections between olfactory and social deficits have been suggested (Malaspina and Coleman, 2003; Lahera et al., 2013), olfaction might also offer original tools to explore interpersonal abilities in alcohol-dependence.

### HOW DOES OLFACTION INTERACT WITH OTHER MODALITIES?

Crossmodal processing is the ability to construct a unified percept on the basis of distinct sensory inputs. This crucial capacity for everyday life has been explored among healthy and psychiatric populations (Campanella and Belin, 2007; De Jong et al., 2009), and is impaired in alcohol-dependence (Maurage et al., 2007, 2008, 2013). However, previous studies focused on audio-visual integration, and it is unclear whether the observed behavioral and cerebral correlates of integration reflect general crossmodal integration or are somehow specific to audio-visual processing. Exploring crossmodal integration between olfactory and visual–auditory stimulations would test the generalization of earlier results to other modalities. Preliminary results exist on olfactory–visual crossmodality, showing that olfactory cues influence emotional visual processing (Leppänen and Hietanen, 2003; Seubert et al., 2010a), while these olfactory–visual influences appear impaired in schizophrenia (Seubert et al., 2010b). Moreover, some cerebral regions (middle frontal gyrus) are activated in all crossmodal situations, while others (anterior insula) seem specifically involved in olfactory–visual interactions (Small, 2004). Applying these paradigms to alcohol-dependence would deepen the understanding of crossmodal processing.

### IS THERE A GLOBAL IMPAIRMENT FOR CHEMICAL SENSES?

Olfaction and taste are intimately linked, notably by the retronasal stimulation (Hornung and Enns, 1987) which appears altered in alcohol-dependence (Maurage et al., 2011b) but has been little explored. Moreover, while links have been shown between gustatory characteristics and risk for alcohol-dependence (Sandstrom et al., 2003), tasting abilities remain surprisingly underexplored. Earlier studies exploring taste in alcohol-dependence indeed focused on sucrose detection, showing preserved (Tremblay et al., 2009) or increased (Kampov-Polevoy et al., 1998) sensitivity to sucrose. Other aspects of taste remain unexplored, despite their role in malnutrition problems. The recent development of validated taste evaluations should lead to the precise exploration of this sensorial modality, in order to complete the description of chemical senses deficits.

### COULD OLFACTION TESTING DETECT COGNITIVE IMPAIRMENT IN ALCOHOL-DEPENDENCE?

Simple olfactory tests have been used to assess cognitive impairments in the early stages of neurological diseases (Moscovich et al., 2012; Conti et al., 2013; Stamps et al., 2013). As it is still currently difficult to detect individuals who are bound to develop alcohol-dependence, these tests might be used to rapidly assess the cognitive decline in alcohol-dependence, and hence serve as early detectors of brain impairments potentially facilitating the maintenance of alcohol abuse.

### WHAT IS THE IMPACT OF OLFACTORY LOSS ON EVERYDAY LIFE IN ALCOHOL-DEPENDENCE?

While it is clearly established that olfaction impairments have a major impact on life satisfaction (Shu et al., 2011) and nutrition (Stevenson, 2010), the precise consequences of olfactory loss on alcohol-dependent individuals’ everyday life remain unexplored. Future studies should thus directly determine how olfaction deficits modulate the quality of life and nutritional habits in alcohol-dependence.

## CONCLUSION

This perspective paper underlined the usefulness of developing a structured and thorough exploration of olfaction in alcohol-dependence. Olfaction is deeply involved in a wide range of everyday life activities (Stevenson, 2010) but olfactory impairments remain largely under-diagnosed and under-treated in healthy and psychiatric populations. Recent findings have underlined the involvement of odor processing impairments in schizophrenia and their usefulness as an endophenotypic marker of vulnerability, which shows that olfaction studies constitute a promising research field to understand this pathology (Rupp, 2010). Accordingly, olfactory cues might be involved in the emergence of alcohol-dependence and in relapse after detoxification. However, data are currently lacking in this field, as earlier studies focused on visual and auditory stimulations.

We tried to show that this lack of data is detrimental for the understanding of alcohol-dependence and to propose several perspectives for the development of this research field. At the fundamental level, further exploring olfaction in alcohol-dependence could on the one hand enrich the knowledge concerning the behavioral and cerebral consequences of excessive alcohol consumption, and thus complement the current theoretical models of this pathology. Specifically, following what has been done in other psychiatric states (Moberg et al., 2013; Schecklmann et al., 2013), olfaction deficits might give crucial new insights on the pathophysiological mechanisms involved in the appearance and maintenance of alcohol-related disorders. On the other hand, these explorations could complement the understanding of the mutual influences between olfaction and cognitive-emotional processes. At the clinical level, circumscribing the extent of olfactory impairments in alcohol-dependence would help clinicians to take into account this deficit and its consequences on everyday life, as it is currently totally neglected in clinical settings. It might also allow the inclusion of olfactory stimulations in crossmodal retraining programs, or even the development of innovative olfaction training programs capitalizing on existing rehabilitation tools used in other populations with olfactory loss (Hummel et al., 2009; Konstantinidis et al., 2013). Actually, nearly everything remains to be done in the exploration of olfaction in alcohol-dependence, but we hope that the preliminary data and research perspectives described here will encourage the development of this research field.

## Conflict of Interest Statement

The authors declare that the research was conducted in the absence of any commercial or financial relationships that could be construed as a potential conflict of interest.
